# A influência da tomografia computadorizada no planejamento pré-operatório da revisão de artroplastia do quadril – Componente femoral

**DOI:** 10.1055/s-0045-1810035

**Published:** 2025-09-08

**Authors:** Rafaela Reis Torrealba, Maria Isabella Cruz de Castro, Phercyles Veiga-Santos, Marcelo Felipe Almeida, Conrado Torres Laett, Lourenço Peixoto

**Affiliations:** 1 Instituto Nacional de Traumatologia e Ortopedia Jamil Haddad, Rio de Janeiro, RJ, Brasil.

**Keywords:** artroplastia de quadril, cirurgia de revisão, cirurgia ortopédica, tomografia computadorizada, arthroplasty, replacement, hip, revision surgery, surgery, orthopedic, tomography, x-ray computed

## Abstract

**Objetivo:**

Este estudo teve como objetivo comparar a acurácia da Classificação de Paprosky de Perda Óssea Femoral utilizando radiografias simples e imagens de tomografia computadorizada bidimensional (TC-2D), com o defeito femoral observado no intraoperatório pelo cirurgião.

**Métodos:**

Um total de 80 pacientes com indicação para a revisão de artroplastia do quadril foram classificados de acordo com Paprosky, por 14 cirurgiões do quadril do mesmo hospital, com base em radiografias simples em anteroposterior da pelve e posteriormente baseadas em TC-2D, reconstruídas nos planos axial, coronal e sagital. Esses dados foram comparados com os achados de perda óssea femoral no intraoperatório pelos mesmos cirurgiões.

**Resultados:**

A concordância entre a avaliação feita por radiografia e TC é excelente para o defeito ósseo femoral (94% de concordância; κ = 0,95; 0,90–0,99). Individualmente, a classificação feita de acordo com a radiografia concorda com a classificação intraoperatória em 85% dos casos (κ = 0,8; 0,70–0,90). A feita com base na TC tem 86% de concordância (κ = 0,84; 0,75–0,93). Não houve diferença estatística entre os métodos.

**Conclusão:**

O uso de TC-2D não mostrou benefícios no reconhecimento da perda óssea femoral pela classificação de Paprosky em comparação com a radiografia. Portanto, deve-se questionar a importância das imagens 2D no planejamento da cirurgia de revisão do componente femoral, visto que se associa com maior custo financeiro e à maior exposição do paciente a níveis elevados de radiação.

## Abstract (en)

**Objective:**

The present study aimed to compare the accuracy of the Paprosky Classification of Femoral Bone Loss using plain radiographs and two-dimensional computed tomography (2D CT) images with the femoral defect observed intraoperatively by the surgeon.

**Methods:**

There were 14 hip surgeons from the same hospital who classified 80 patients with an indication for revision hip arthroplasty according to Paprosky based on plain radiographs in anteroposterior views of the pelvis and 2D CT images, reconstructed in the axial, coronal, and sagittal planes. We compared this data with the intraoperative findings of femoral bone loss by the same surgeons.

**Results:**

The agreement between the radiographic and CT assessment was excellent for femoral bone defects (94% agreement; κ = 0.95; 0.90–0.99). Individually, the radiograph-based classification agreed with the intraoperative classification in 85% of cases (κ = 0.8; 0.70–0.90). The CT-based one had 86% of agreement (κ = 0.84; 0.75–0.93). There was no statistical difference between the methods.

**Conclusion:**

The use of 2D CT did not show any benefits in recognizing femoral bone loss by the Paprosky classification compared with radiography. Therefore, the significance of 2D images in planning femoral component revision surgery should be questioned, as it is associated with higher financial costs and greater patient exposure to high radiation levels.

## Introdução

A artroplastia total do quadril (ATQ) é um procedimento extremamente eficaz para o tratamento de artrose do quadril. Com o aumento do número de ATQs realizadas, a diminuição da idade dos pacientes operados e o aumento da expectativa de vida, o número de cirurgias de revisão tem aumentado. Espera-se que a necessidade da cirurgia vai superar 130% até 2030. 1 2 3

As indicações para revisão da ATQ (rATQ) incluem osteólise, instabilidade, soltura asséptica, fraturas, infecções e desgaste. 3 4 Em diferentes graus, todas as causas para revisão cursam com perda óssea. Devido à alta complexidade nesse tipo de cirurgia, o planejamento pré-operatório deve ser minucioso.

Por meio da classificação de Paprosky, com base na avaliação por imagem no pré- ou no intraoperatório, o cirurgião é capaz de fazer o planejamento do melhor implante e técnica a serem utilizadas. 5 6 Essa classificação para perda óssea femoral foi desenvolvida em 1994 e contempla quatro estágios progressivos de defeito. O tipo I corresponde a um defeito metafisário mínimo, o II a um extenso defeito metafisário e com mínima perda diafisária. Paprosky tipo III é dividido em A e B, com o IIIA correspondendo a um extenso defeito metafisário e diafisário, além da diáfise estar intacta até ≥ 4cm do ístmo; enquanto o tipo IIIB representando um extenso defeito metafisário e diafisário e a diáfise intacta até < 4 cm do ístmo. Os defeitos tipo IV são os mais graves e representam um extenso defeito metafisário e diafisário com o ístmo insuficiente. 6 Há uma extensa documentação na literatura de que radiografias simples (raios-X) e tomografia computadorizada (TC) garantem precisão e reprodutibilidade e minimizam complicações intraoperatórias, melhorando a taxa de sucesso da cirurgia de revisão.

Este estudo teve como objetivo comparar a acurácia da classificação de Paprosky utilizando radiografias simples e imagens de TC bidimensionais (2D), com o defeito femoral observado no intraoperatório pelo cirurgião. Nosso objetivo é garantir um planejamento pré-operatório da cirurgia de revisão do componente femoral de forma segura e tecnicamente reprodutível.

## Materiais e Métodos

Um total de 80 pacientes com indicação de revisão de artroplastia do quadril foram acompanhados neste estudo observacional e prospectivo entre janeiro de 2021 e dezembro de 2022. Os critérios de inclusão para os pacientes foram idade superior a 30 anos e radiografia evidenciando falha asséptica do componente femoral. Aqueles com fratura periprotética ou infecção ativa foram excluídos.

Todos os pacientes foram avaliados de acordo com a classificação de Paprosky ( Tabela 1 ) por 14 cirurgiões sênior (> 10 anos) e júnior (< 10 anos), membros do Centro Especializado do Quadril do nosso hospital, com base em imagens de radiografia simples em anteroposterior da pelve e, posteriormente, com base em TC-2D, reconstruídas nos planos axial, coronal e sagital. As imagens utilizadas para análise foram extraídas do arquivo médico do hospital, pelo programa MDICON.

**Tabela 1:** Classificação de Paprosky de perda óssea femoral

Tipo	Descrição
I	Defeito metafisário mínimo
II	Extenso defeito metafisário, com mínima perda diafisária
IIIA	Extenso defeito metafisário e diafisário, diáfise intacta até ≥ 4 cm do istmo
IIIB	Extenso defeito metafisário e diafisário, diáfise intacta até < 4 cm do istmo
IV	Extenso defeito metafisário e diafisário, istmo insuficiente

O planejamento cirúrgico contemplou a avaliação do implante femoral mais adequado a ser utilizado, necessidade do uso de enxerto ósseo e a presença ou não de descontinuidade pélvica. Uma nova avaliação foi feita 30 dias após a primeira, seguindo os mesmos critérios de classificação e planejamento cirúrgico. A ordem das imagens foi alterada aleatoriamente por meio do programa Excel (Microsoft Corp.) 2019. Não houve identificação dos prontuários nem dos nomes dos pacientes. A classificação da perda óssea femoral observada no intraoperatório foi considerada padrão-ouro.

Todos os cirurgiões e pacientes assinaram o termo de consentimento livre e esclarecido (TCLE). O estudo foi aprovado pelo Comitê de Ética em Pesquisa sob o parecer n° 003760/2021 e CAAE n° 42181421.5.0000.5273.

A concordância entre as classificações foi expressa em termos percentuais e avaliada pelo coeficiente kappa (κ), sendo separada em ruim (< 0,20), fraca (> 0,20 e < 0,40), moderada (> 40 e < 0,60), boa (> 60 e < 0,80) e excelente (> 0,80), com um IC de 95% apresentado por Landis e Koch. 7 A comparação entre os valores de κ foi feita por meio do IC, sendo considerado estatisticamente diferente quando não há sobreposição.

## Resultados

A concordância entre a avaliação feita por radiografia e TC é excelente para o defeito ósseo femoral (94% de concordância; κ = 0,95; 0,90–0,99), como pode ser visto na Tabela 2 .

**Tabela 2:** Matriz de confusão para classificação de defeito ósseo femoral

		Tomografia computadorizada				
		1	2	3A	3B	4
**Radiografia**	**1**	28	0	0	0	0
	**2**	0	29	2	0	0
	**3A**	0	0	20	1	0
	**3B**	0	0	0	8	0
	**4**	0	0	0	0	2

**Fig. 1:**
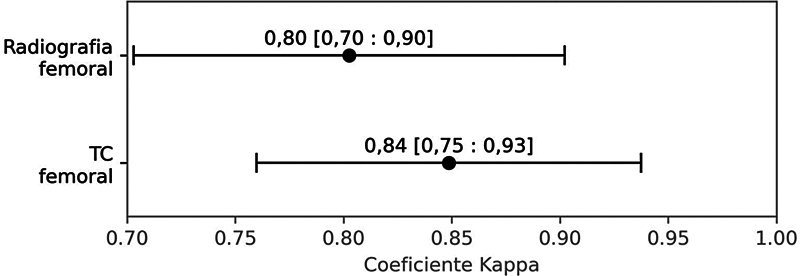
Valores do coeficiente kappa (IC de 95%) para avaliação da concordância na classificação de Paproski para defeitos ósseos do quadril. **Abreviatura:** TC, tomografia computadorizada.

A classificação feita pela radiografia concorda com a classificação intraoperatória em 85% dos casos. A TC demonstrou 86% de concordância. Não foi observada diferença estatística entre os métodos ( Fig. 1 ).

O planejamento cirúrgico realizado por radiografias e TCs concordam plenamente. Observou-se concordância de 90% entre o planejamento cirúrgico e o procedimento realizado no componente femoral (κ = 0,74; 0,59–0,89).

## Discussão

A classificação de Paprosky é uma das ferramentas que auxilia no planejamento pré-operatório da cirurgia de revisão da ATQ. Além disso, é capaz de quantificar a perda óssea do componente femoral com base na imagiologia e escolher o melhor implante e técnica a ser utilizada. Assim, a classificação dos defeitos ósseos predeterminados deve ser fiel ao que o ortopedista encontrará no intraoperatório.

Para que uma classificação seja útil, ela deve apresentar boa confiabilidade inter- e intra-observador. Brown et al. 8 reuniu quatro cirurgiões de quadril para avaliar a perda óssea femoral pela classificação de Paprosky de 205 radiografias de pacientes antes da revisão. Essas imagens foram exibidas aleatoriamente um mês depois para todos os cirurgiões as classificarem novamente. Encontrou-se confiabilidade interobservador substancial (0,61) e concordância intraobservador consistente (0,81, 0,78, 0,76 e 0,75).

O objetivo do nosso estudo é analisar o valor preditivo da classificação de Paprosky baseada na radiografia simples e na TC-2D, para o planejamento pré-operatório. Na prática, um alto valor preditivo possibilita planejar e escolher o implante que provavelmente será utilizado e o tipo ou quantidade de enxerto ósseo, se necessário. 9 Confirmamos que a classificação de Paprosky para perda óssea femoral tem concordância substancial quando o que foi planejado no pré-operatório foi encontrado e realizado no intraoperatório. No entanto, não encontramos uma diferença importante entre os modelos de raio-X e TC-2D.

A literatura limita-se em artigos que avaliem a acurácia do planejamento pré-operatório utilizando raio-X e TC-2D em casos de perda óssea femoral. Até a presente data, a maioria dos autores avaliou os dados supracitados em cirurgias primárias de artroplastia total do quadril. Muitos estudos já comprovaram que modelos de TC tridimensional (3D) associam-se a uma excelente confiabilidade em relação ao tamanho e alinhamento dos componentes planejados. 10 11 12 Sariali et al. 13 e Reinbacher et al. 14 compararam o uso de modelos 2D versus 3D para definir o tamanho da copa e da haste femoral a serem implantados e observaram superioridade na eficiência, acurácia e reprodutibilidade dos moldes em 3D.

No entanto, a literatura ainda é muito escassa no âmbito do planejamento das cirurgias de revisão e ainda mais para a revisão do componente femoral. A vasta maioria inclusa aqui vislumbra a revisão do componente acetabular. Winter et al. 15 avaliaram a precisão do planejamento pré-operatório através das impressões em 3D, quando comparado a 2D, em 27 pacientes que necessitavam de gaiolas de reforço acetabular (Burch-Schneider). O tamanho dos implantes planejados foram comparados com os tamanhos finais. Os moldes customizados em 3D previram o tamanho exato do implante em 96,3% dos pacientes, comparado a apenas 55,6% do planejamento em 2D. Embora isso sugira fortemente que as reconstruções 3D são mais confiáveis, é importante destacar que os pacientes incluídos apresentavam defeitos ósseos maciços, categorizados como Paprosky tipo IIA-IIIB, e aqueles com menor perda óssea foram excluídos. Além disso, o estudo não deixou claro o nível de experiência do cirurgião.

Plate et al. 16 compararam a classificação de Paprosky para perda óssea acetabular de 8 revisões de ATQ com base em radiografias simples, TC-2D e reconstruções em 3D. As imagens foram mostradas a 35 residentes do primeiro ano de treinamento da pós-graduação, 2 fellows e 2 cirurgiões ortopédicos, cujas respostas foram comparadas com as dadas por 2 cirurgiões de quadril experientes. Afirmou-se que o raio-X levou a um aumento do número de classificações corretas (0,37), enquanto a TC-2D e as reconstruções em 3D não melhoraram a precisão (0,33, 0,20; *p* < 0,001), o que contradiz a grande maioria dos estudos existentes. Os autores ressaltam que o nível de experiência dos cirurgiões não influenciou na classificação correta de Paprosky.

Alguns autores avaliaram a confiabilidade de outros sistemas de classificação de perda óssea baseados em radiografias. Käfer et al. 9 contaram com dois cirurgiões de quadril para avaliarem a radiografia de 33 pacientes quanto ao defeito ósseo presente e decidirem o melhor implante e técnica para cada caso. Outro examinador foi responsável por verificar se o procedimento planejado seria realizado pelo cirurgião. A classificação utilizada foi a de Saleh. A análise entre as estimativas radiológicas pré-operatórias e as conclusões intraoperatórias referentes ao implante e ao enxerto ósseo revelou coeficientes de correlação de 0,63 ( *p* < 0,01) para a classificação femoral. Pode-se argumentar que a diferença encontrada entre o uso da classificação de Paprosky e Saleh é que o primeiro afirma que se deve considerar o defeito radiográfico visível e o provável a ser encontrado após a remoção do implante. Além disso, o número de pacientes que participaram deste estudo é menor do que o nosso.

Algumas limitações foram constatadas no presente estudo. Classificamos os cirurgiões de quadril que contemplam menos de 10 anos de experiência como júnior e os com mais de 10 anos classificamos como senior. Acreditamos que o tempo de experiência possa interferir na acurácia da classificação de Paprosky tanto no pré- quanto no intraoperatório. Apesar de comprovarmos que o planejamento pré-operatório com o auxílio da TC-2D não apresenta benefícios em relação à radiografia, é sabido que modelos 3D são considerados padrão ouro. Entretanto, esse recurso não foi utilizado para base de comparação.

## Conclusão

A utilização da TC-2D não mostrou benefícios no reconhecimento da perda óssea femoral pela classificação de Paprosky. Além disso, essa técnica de imagem, nesses casos, está associada a custos mais elevados, em comparação à radiografia e a maior exposição do paciente a elevados níveis de radiação. Portanto, deve-se questionar a importância desse recurso assistencial no planejamento do componente femoral na cirurgia de rATQ.
